# Beyond Removal: A Critical Review of Microplastic Mass Flux, In-Plant Transformation, and Elimination in WWTPs

**DOI:** 10.3390/molecules31050798

**Published:** 2026-02-27

**Authors:** Niu Imeleta Faauma, Ying Guo, Wenxin Li, Wei Wen, Bo Jiang

**Affiliations:** 1 School of Energy and Environmental Engineering, University of Science & Technology Beijing, Beijing 100083, China; imefitiao@gmail.com (N.I.F.); gy18531861867@163.com (Y.G.); m202320220@xs.ustb.edu.cn (W.L.); 2 National Engineering Laboratory for Site Remediation Technologies, Beijing 100015, China

**Keywords:** microplastics, wastewater treatment plants, nanoplastics, mass flux analysis, polymer mineralization, sewage sludge partitioning, advanced oxidation processes (AOPs), dual-metric framework

## Abstract

Microplastics (MPs) persist in wastewater treatment systems owing to their durability and mobility. As critical interception points, wastewater treatment plants (WWTPs) receive MPs from diverse domestic and industrial sources. This review synthesizes peer-reviewed studies (2009–2026) to evaluate MP mass flux, in-plant transformation, and elimination across primary, secondary, and tertiary stages. While conventional processes typically remove 60–90% of MPs, advanced tertiary technologies, such as membrane bioreactors and rapid sand filtration, can achieve efficiencies exceeding 95%. The fate of MPs is governed by density-driven settling and biological aggregation; however, the significant accumulation of MPs in sewage sludge represents a critical pathway for environmental re-entry. This review highlights key knowledge gaps, including inconsistent analytical methodologies, evidence of in-plant fragmentation generating nanoplastics (NPs), and uncertainties regarding full-scale mass flows. Furthermore, the review synthesizes mass flux data to clarify the partitioning of MPs between the effluent and sludge, identifying biosolids as a primary sink. The review concludes by proposing a transition from physical separation to elimination technologies (e.g., AOPs), alongside standardized monitoring and regulatory frameworks, to achieve sustainable reductions in MP emissions.

## 1. Introduction

Plastic fragments smaller than five millimeters, collectively termed microplastics (MPs), have become a pervasive global pollutant. Detected across nearly all environmental compartments, from the atmosphere to deep-sea sediments, MPs originate from two main categories: primary MPs, intentionally manufactured for industrial or consumer use (e.g., cosmetic microbeads and resin pellets), and secondary MPs, formed through the weathering and fragmentation of larger plastic debris [1,2]. These pollutants pervade aquatic and terrestrial ecosystems, posing significant risks to environmental health and acting as vectors for heavy metals and persistent organic pollutants that can be transferred through food webs to humans [3].

As the primary interface between anthropogenic waste and the environment, wastewater treatment plants (WWTPs) have emerged as critical focal points for microplastic (MP) management. Although WWTPs are designed to treat organic matter and nutrients, they function as unintentional capture points for MPs originating from domestic laundering, industrial effluents, and urban runoff [4,5]. While conventional treatment processes can capture a significant fraction of influent MPs, studies indicate that considerable quantities still escape via the treated effluent [5,6]. In addition, the removal of MPs from the liquid phase often results in their accumulation in sewage sludge, creating a secondary contamination pathway when biosolids are applied to agricultural land. This ongoing release underscores the urgent need for improved treatment technologies and coordinated management frameworks to reduce the environmental footprint of wastewater infrastructure [2,7].

Despite the growing body of literature on this topic, significant knowledge gaps remain. Previous reviews have largely focused on physical removal efficiencies in isolation without considering internal dynamics, often overlooking the internal mechanisms of in-plant fragmentation and the subsequent partitioning of plastics into sewage sludge, which creates a secondary contamination pathway in terrestrial environments. However, the complexity of MP behavior, including size-dependent removal, in-plant fragmentation, and phase partitioning, requires a more holistic assessment, a perspective often limited in prior, more siloed studies. Correspondingly, emerging biological strategies, such as enzymatic degradation, show promise in laboratory settings but face significant scalability challenges before they can be effectively integrated into operational WWTPs [8]. Additionally, there is a critical need for standardized sampling and analytical methods, as their absence currently hinders a comprehensive understanding of MP transformation and its long-term effects in wastewater systems [4,6].

To address these gaps, this review integrates three complementary dimensions of microplastic behavior. First, we analyzed mass flux dynamics, integrating previously disparate data to clarify the partitioning of pollutants between the effluent and sludge. Second, we examined in-plant transformation, specifically the fragmentation of MPs into nanoplastics (NPs) that bypass conventional barriers and thus pose an underestimated environmental risk. Finally, we evaluated the transition from physical separation to polymer mineralization, thereby proposing a novel framework for next-generation treatment strategies that focus on reducing toxicity. Furthermore, by evaluating the discrepancies between current analytical protocols, this study proposes a dual-metric framework to overcome inconsistencies in reporting and enhance the detection of smaller, often-overlooked plastic fractions.

## 2. Methodology and Data Collection

To critically evaluate the current state of mass flux, transformation, and elimination of MPs in WWTPs, a systematic literature review was conducted. Peer-reviewed articles, technical reports, and official government documents published between 2009 and 2026 were systematically identified using major scientific databases (Web of Science, Scopus, and Google Scholar). The 2009 start date corresponds to the emergence of standardized microplastic research in wastewater, while the inclusion of technical and government documents aimed to capture broader policy frameworks and practical operational insights that complement academic research.

The search strategy used Boolean operators (AND and OR) to combine core keywords with specific phrases related to quantitative outcomes and process mechanisms. This approach prioritized studies that reported quantitative data on (i) full-scale mass balances and partitioning of MPs between treated effluent and sewage sludge; (ii) in-plant transformation processes, including fragmentation and the formation of NPs; and (iii) removal efficiencies of advanced treatment technologies (e.g., membrane bioreactors and advanced oxidation processes) and their potential to achieve polymer mineralization.

Search terms included combinations of “microplastics,” “nanoplastics,” “wastewater treatment plants,” “mass flux,” “sludge accumulation,” “in-plant transformation,” and “toxicity of degradation intermediates.” Records were initially screened by title and abstract, followed by a full-text review to verify methodological relevance and data quality. A total of 141 studies were selected for synthesis. To ensure global applicability, the review included data from diverse geographic regions, treatment configurations, and plant scales, while studies focusing solely on small-scale laboratory tests were excluded unless they provided essential mechanistic insight, specifically those elucidating novel degradation pathways, transformation processes, or toxicity effects not observable at larger scales.

## 3. Sources and Characteristics of MPs in WWTP

Global plastic production surpassed 400 million tons by 2020, reflecting the sustained growth trajectory outlined and corroborated by recent production statistics reported by Plastics Europe [1,9,10]. As these materials enter wastewater streams, they exhibit diverse shapes and physical traits that fundamentally influence their removal and fate.

Table 1 outlines the morphological classification and physical characterization of these MPs, establishing the key parameters used to evaluate their behavior throughout the treatment process.

### 3.1. Primary MPs: Sources and Entry Pathways

Primary MPs are known to produce polymer particulates, usually under five millimeters [2,4], engineered for specific industrial and commercial applications (Table 2), such as introduction into the environment through industrial discharges, consumer product use, and inefficient waste management practices. Because of their small dimensions and chemical resilience, primary MPs display strong mobility across environmental compartments, bypassing conventional filtration systems in WWTPs. Their prevalence stresses the need for wide-ranging research on their sources, pathways, and ecological impacts to inform effective mitigation strategies [2].

### 3.2. Secondary MPs: Origins from Weathering and Fragmentation

Secondary MPs originate from the gradual degradation of larger plastic materials through ultraviolet exposure, mechanical abrasion, and chemical weathering [7,12]. These processes yield particles such as textile fibers released during laundering, tire-wear particles, fragments from deteriorating paints, and debris from aquatic equipment, including fishing nets and ropes [13], as summarized in Table 3. Typically, <5 mm and sometimes as small as 1 µm [7,11], these irregularly shaped particles have diverse polymer compositions, which contribute to their high surface reactivity and their ability to adsorb co-occurring pollutants. In WWTPs, secondary MPs can impair treatment performance by disrupting microbial processes and accumulating in sludge [13]. While treatment systems remove a substantial portion of these particles, a fraction nonetheless passes through the treated effluents, serving as an ongoing pathway for MP discharge into the environment [4,13].

### 3.3. Morphological Classification and Size Spectrum

**Figure 1:**
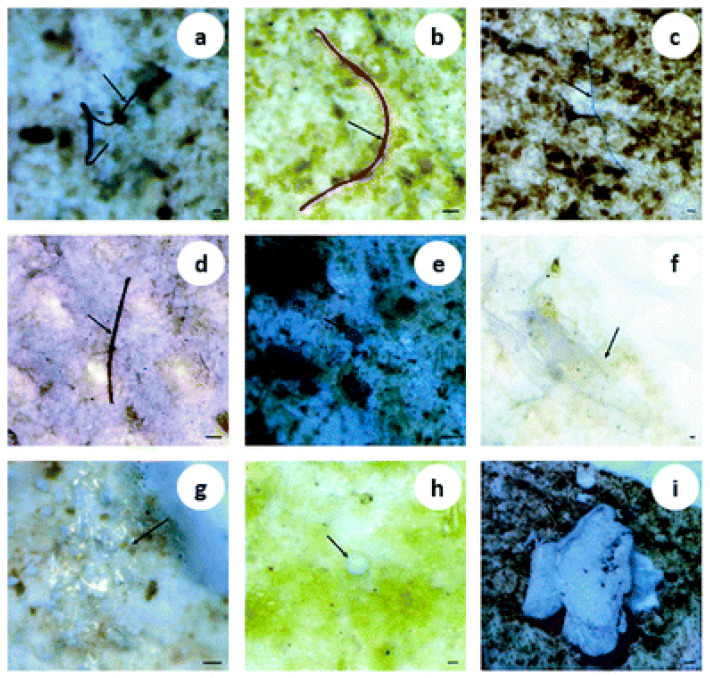
Representative Morphological Types of MPs Identified in Environmental Samples: (**a**–**d**) fibers; (**e**,**f**) fragments; (**g**) films; (**h**,**i**) granules. The colors represent the natural appearance of the particles and the surrounding environmental matrix under optical microscopy. Scale bar = 100 µm. Reproduced from ref. [28] with permission from the Royal Society of Chemistry.

Representative micrographs show common MP morphologies observed in environmental samples (Figure 1). These images illustrate the typical MP forms documented across environmental matrices and are not specific to MPs isolated from WWTPs.

### 3.4. Physicochemical Properties Governing Transport and Fate

The physical and chemical characteristics of MPs, including polymer density, surface morphology, and crystallinity, are fundamental determinants of their behavior throughout the treatment process. These properties influence how particles interact with chemical coagulants and biological flocs, directly affecting their velocity and settling rates. Table 4 summarizes the diverse properties of MPs identified across various wastewater treatment stages. Ultimately, these intrinsic physical and chemical properties dictate the subsequent entry mechanisms and complex partitioning behavior. These fundamental characteristics serve as the basis for the mass flow dynamics and environmental fate analyses detailed in Section 3.

## 4. Occurrence, Partitioning, and Mass Flux Dynamics in WWTPs

### 4.1. Influent Loading and Transport Pathways

MPs enter wastewater treatment facilities through a complex network of conveyance systems and are primarily categorized into domestic discharge, industrial effluent, and urban runoff. Understanding these entry pathways is critical because they determine the hydraulic pulse and particle concentration peaks that WWTPs must manage.

Unlike the consistent loading observed from domestic sources, inputs from combined sewer systems (CSS) differ significantly from separate sewer systems (SSS). In the CSS, precipitation events mobilize terrestrial MPs, such as tire wear particles (TWPs) and road dust, delivering high-intensity “first flush” loads that can bypass treatment during overflow events [40,41]. Conversely, industrial lines often introduce specific polymer types (e.g., pellets or pre-production resins) in distinct pulses linked to manufacturing cycles [42], as summarized in Table 5.

**Figure 2:**
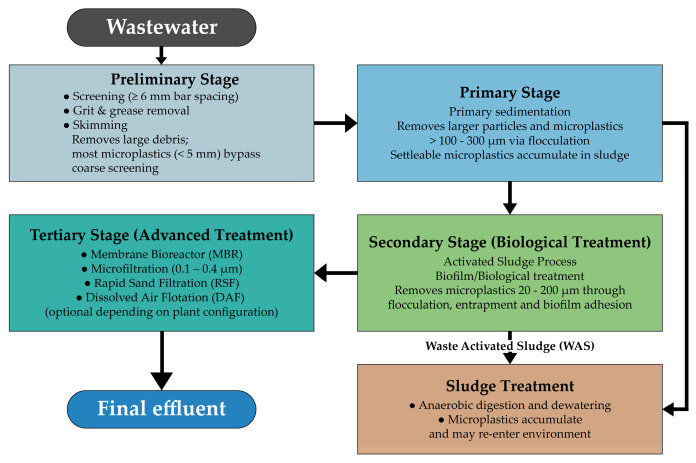
MP Pathways and Size-Dependent Separation and Sludge Partitioning in a Conventional WWTP.

Conceptual diagram illustrating MP pathways and removal mechanisms across the treatment stages of a conventional WWTP (Figure 2). Preliminary screening (bar spacing ≥ 6 mm) and grit-removal units primarily capture large debris, thereby allowing most MPs (<5 mm) to bypass these stages [6]. During primary sedimentation, density-driven settling and flocculation promote the removal of larger or more settleable MPs, typically > 100–300 µm [4]. Secondary biological treatment further reduces MP loads through bio-flocculation, entrapment within activated-sludge flocs, and biofilm adhesion, particularly for particles in the 20–200 µm range; however, buoyant or fibrous MPs may persist through this stage [4,6]. Tertiary and advanced treatment technologies—including rapid sand filtration, dissolved air flotation, microfiltration/ultrafiltration (≈0.1–0.4 µm), and membrane bioreactors—provide enhanced physical retention and can achieve removal efficiencies exceeding 97–99% [50]. In addition to effluent discharge pathways, the diagram also depicts the accumulation of MPs in primary and secondary sludge, which serves as a major sink within WWTPs and a significant vector for environmental re-entry during sludge treatment, land application, and disposal [51,52]. These pathways highlight the dual role of WWTPs as barriers and conduits for MP dissemination across aquatic and terrestrial environments.

- Preliminary Treatment (screening):

Preliminary treatment provides the first barrier to particulate pollutants entering a wastewater treatment plant. Although its primary purpose is to remove coarse materials, it also measurably reduces MPs [6,13]. Mechanical screening and grit-removal units intercept larger plastic fragments and fiber clusters, which are physically retained by bar racks, mesh screens, and sedimentation channels [6]. Operational observations from full-scale facilities indicate that a notable fraction of influent MPs is already diminished at this initial stage, with fibrous particles particularly prone to capture owing to their elongated morphology and tendency to intertwine with larger debris and settleable solids [6,40]. Consequently, these findings highlight that even before primary clarification, preliminary treatment can meaningfully reduce the MP burden entering subsequent treatment processes, thereby influencing the overall system performance [53].

- Primary Treatment (sedimentation):

Primary treatment is a critical phase in MP removal, relying on gravitational settling and density stratification to separate particulate matter from incoming wastewater. During this stage, MPs with higher densities or those associated with organic aggregates tended to settle preferentially in the primary sludge. Simultaneously, lighter or more buoyant polymers remain suspended and advance to subsequent treatment. Observations from full-scale urban facilities confirm this pattern [13,54]; specifically, Sun et al. [6] and Bayo et al. [54] reported a marked reduction in MP concentrations following primary clarification, with particle morphology and local physicochemical parameters strongly influencing removal performance. In particular, fragments and heavier particles exhibited greater settling tendencies, whereas fibers and buoyant polymers were more likely to escape the clarifier. These findings highlight the importance of sedimentation dynamics in shaping the shape of MP during the early stages of wastewater treatment.

- Secondary Treatment (Biological Treatment):

Secondary treatment plays a pivotal role in shaping the fate of MPs in wastewater treatment systems. In activated sludge and other biological processes, MPs may be incorporated into microbial flocs or immobilized within developing biofilms, where extracellular polymeric substances contribute to their temporary retention. Despite these interactions, MPs are resistant to biodegradation and therefore remain structurally intact throughout biological processing. Reported removal efficiencies for conventional secondary units vary widely, primarily reflecting differences in operational conditions, sludge characteristics, and particle morphology; in particular, fibers often evade capture owing to their buoyancy and limited affinity for settling flocs. Nonetheless, biological treatment substantially reduces the MP load entering tertiary units, and advanced biological systems, such as membrane bioreactors, have demonstrated markedly higher retention owing to their fine physical separation barriers. Collectively, these findings highlight the importance of secondary treatment as an intermediate but incomplete barrier to MP release [51,52].

- Tertiary Treatment (Filtration and Disinfection):

Sand Filtration: Filtration represents a practical approach for removing specific categories of MPs from wastewater, particularly microfibers and particles larger than the pore size of the filter. Tertiary filtration systems may incorporate fine-mesh screens, rapid sand filters, or membrane-based technologies such as microfiltration and ultrafiltration. Fine-mesh units with small pore openings can physically retain larger MPs, whereas granular media beds capture particles through straining, interception, and depth filtration [53,54]. These approaches are beneficial for removing fibers and irregular fragments that are commonly discharged from household laundry and industrial effluents [55]. However, their efficiency markedly decreases for smaller MPs and NPs, which readily penetrate conventional filter media and require advanced membrane processes for reliable removal [51,52,55].

Rapid Sand Filtration (RSF):

This tertiary polishing technique is widely applied in modern wastewater treatment systems. This process relies on the passage of secondary effluents through a granular sand bed, where MPs are retained by physical straining, interception, and depth filtration. RSF is particularly effective at capturing MPs larger than several tens of micrometers, including elongated fibers and irregular fragments that become lodged within the filter media. The reported removal efficiency for the RSF approach was 97%. However, the actual performance can vary with factors such as media grain size, filter depth, hydraulic loading rate, and particle characteristics in the influent. Owing to its operational simplicity and compatibility with existing treatment infrastructure, RSF is a practical and robust option for improving MP removal during tertiary treatment [50].

UV disinfection is ineffective in removing MPs because it targets microorganisms rather than particulate contaminants. The MP concentrations remained unchanged after UV treatment because UV provides no physical separation mechanism for solid particles [51,52].

Despite the high removal efficiencies reported for membrane-based processes, recent evidence indicates that MPs can interfere with their operation by accelerating membrane fouling. In tertiary wastewater treatment, MP particles have been shown to accumulate on membrane surfaces, obstruct pore openings, and promote the development of compact cake layers, thereby increasing hydraulic resistance and reducing filtration capacity [56]. Mechanistic studies on ultrafiltration membranes further revealed that both nano- and MP particles can adsorb onto pore walls, constrict pore channels, and increase transmembrane pressure, eventually contributing to rapid performance decline [57]. These findings align with the broader observations summarized in recent reviews, which note that microplastic-induced fouling not only diminishes permeate flux but also raises energy demand and maintenance frequency, posing substantial operational challenges for long-term membrane performance [58]. Overall, although membrane technologies remain effective for MP removal, their susceptibility to microplastic-related fouling emphasizes the need for careful consideration in the design, operation, and optimization of advanced treatment processes.

- Sludge Treatment:

Sludge management plays a crucial role in controlling the fate of MPs in WWTPs because a large share of particles is removed during primary and secondary processes in sewage sludge. During sedimentation and bioflocculation, MPs readily bind with organic matter and biomass, helping their transfer into primary sludge and waste-activated sludge, where they remain due to their chemical stability and resistance to biodegradation. Studies have shown that standard sludge treatment methods, including anaerobic digestion and mechanical dewatering, do not effectively degrade or remove MPs, leaving these particles largely intact during processing and handling [52]. Therefore, the reuse or disposal of biosolids is a key pathway for environmental redistribution, especially when treated sludge is applied to agricultural soils. Land application can lead to long-term accumulation of MPs in terrestrial environments. It may also allow re-entry into freshwater systems via runoff, erosion, or leaching, reinforcing the need for improved sludge treatment and management practices to lower secondary pollution risks [51]. Overall, these findings highlight the importance of incorporating microplastic-specific considerations into sludge treatment strategies to reduce persistence and ecological impact.

To systematically evaluate how individual treatment stages reduce MP contamination, Table 6 summarizes the published removal efficiencies across primary, secondary, and advanced wastewater treatment processes. The table outlines the particle size ranges targeted by each unit operation, the main physical and biological mechanisms responsible for capturing MPs, and the variability observed among full-scale and pilot-scale systems. This comparative summary provides a structured framework for assessing the relative effectiveness of conventional and advanced treatment technologies. This highlights the ongoing challenges posed by small, buoyant, or morphologically diverse MPs that are not adequately removed in current wastewater treatment systems.

### 4.2. Mass Balance Analysis and Fate Partitioning

Building on this stage-wise perspective, recent full-scale and pilot-scale studies have extended the analysis to plant-wide mass flows, quantifying how MPs and NPs partition between the treated effluent and sewage sludge along the treatment train. This shift moves beyond simple concentration reporting towards flux-based assessments of MPs in WWTPs, enabling a more quantitative evaluation of process-dependent fate and partitioning [61,62,63,64]. These studies typically integrated stage-resolved particle counts or mass concentrations with hydraulic loading rates to estimate influent loads, intra-plant redistribution, and net emissions via treated effluent and sewage sludge [61,62,64,65]. Such mass-balance approaches reveal that nominal “removal efficiencies” derived from influent–effluent concentration differences can obscure substantial internal recycling, accumulation in sludge, and size-selective retention, which collectively govern the true environmental fluxes of MPs and NPs [4,61,62,65].

Moreover, experimental data from municipal WWTPs operated with conventional activated sludge and advanced configurations demonstrate pronounced gradients in both number-based and mass-based loads along the treatment train [61,64,65,66]. Mass-resolved measurements using thermoanalytical techniques have shown that total MP mass concentrations can decrease by more than one order of magnitude from influent to final effluent, while a substantial fraction of the incoming plastic mass is progressively transferred into primary and waste activated sludge streams [61,62,65]. Parallel monitoring campaigns that couple influent and effluent concentrations with plant-specific flow data report daily discharges of the order of 10^8^–10^11^ particles per day from single facilities, despite overall removal efficiencies frequently exceeding 85–90% when expressed on a concentration basis [4,63,64,65]. These findings highlight that even relatively low residual concentrations in effluents can translate into substantial annual fluxes to receiving waters when multiplied by large treatment volumes [4,64,65].

Mass-flow analyses also provide critical insights into the relative importance of sludge as a long-term reservoir and secondary emission pathway for MPs [62,63,65]. Several recent syntheses indicate that the majority of MP inputs—often exceeding 65–80% of the influent load—are finally sequestered in primary and secondary sludge, where they can reach concentrations of tens to hundreds of particles per gram dry weight, depending on the plant configuration and sludge treatment regime [63,64,65,66]. This preferential retention of denser fragments and biofilm-coated particles in sludge contrasts with the enrichment of finer size fractions and buoyant polymers in the effluent, thereby creating distinct flux signatures for the aqueous and solid lines [63,65]. When these biosolids are applied to agricultural soils or disposed via landfilling, the accumulated MPs can be gradually remobilized into terrestrial runoff and atmospheric pathways, reinforcing the role of WWTPs as sinks and delayed sources within the broader MP cycle [62,63,65].

A notable outcome of recent stage-resolved monitoring is the identification of strong size-dependent and morphology-dependent trends in removal and fragmentation behaviors that complicate mass-balance closure [4,65]. In large-scale plants serving megacities, the removal of coarse MPs larger than approximately 1 mm during primary clarification can exceed 85–90%, whereas the apparent removal of sub-200 µm particles is markedly lower and, in some cases, their relative proportion increases after secondary treatment, suggesting in-plant fragmentation and shear-induced generation of finer debris [4,61,65]. At the same time, the predominance of small, irregular fragments and fibers in secondary sludge and waste streams indicates that hydrodynamic conditions, flocculation dynamics, and membrane retention in membrane bioreactor systems jointly shape the observed mass flow patterns across unit operations [64,65]. These size-selective processes complicate the extrapolation of laboratory-scale removal data to full-scale fluxes, highlighting the need for harmonized analytical protocols that capture both micro- and nanosized fractions [4,61,64].

Despite these advances, significant uncertainties remain in closing the MP mass balances at the WWTP scale [61,62,65]. Meta-analyses of influent, effluent, and sludge datasets suggest that only a small percentage of the MPs removed from the water line can be explicitly accounted for in biosolids when relying solely on conventional particle-counting approaches, implying that degradation, transformation to NPs, sampling artefacts, and methodological detection limits may collectively account for large “missing” fractions [61,62,65]. In addition, short-term grab sampling can underrepresent the temporal variability driven by combined sewer overflows, storm events, and diurnal loading patterns, leading to biased estimates of both instantaneous and annual fluxes [4,64,65]. Addressing these knowledge gaps will require the integration of standardized, mass-based analytical protocols, long-term composite sampling, and coupled hydrodynamic–particle tracking models capable of resolving mass flows across individual treatment units and into downstream environmental compartments [4,64,65].

## 5. Analytical Methods for MP Detection and Identification

To address the critical need for methodological standardization identified in the introduction, this section evaluates the technical limitations of current analytical protocols and details how an integrated reporting framework can minimize existing data inconsistencies. Accurate detection and quantification of MPs in WWTPs are critical for assessing removal efficiencies and understanding environmental release. However, the complexity of wastewater matrices, rich in organic matter and suspended solids, poses significant challenges for their isolation and identification.

Current analytical protocols typically follow a multistep workflow involving sampling, pretreatment, and instrumental analysis. Standardized extraction procedures, such as density separation and wet peroxide oxidation (WPO), are essential for isolating plastic particles from biogenic interference. Following extraction, advanced spectroscopic and thermal techniques allow for the definitive identification of polymer types, particle size distributions, and morphological characteristics. Table 7 summarizes the methodological framework currently used to characterize MPs in wastewater matrices.

### 5.1. The Mass-Number Dichotomy and Detection Limits

Despite these standardized workflows, a fundamental challenge remains in the mass-number dichotomy [11,51]. While spectroscopic methods such as μ-FTIR and Raman microscopy provide high-fidelity particle counts and polymer identification [70], their spatial resolution limits (typically > 10–20 μm) result in a critical “analytical blind spot” [69,71]. As a result, these techniques often fail to detect the sub-10 μm fraction, which is the precise size range generated by in-plant fragmentation (Section 6).

Conversely, thermo-analytical methods such as Pyrolysis-GC/MS quantify the total polymer mass (mg/L) without size limitations but sacrifice morphological data [68,73]. This methodological disconnect leads to a significant underestimation of the nanoplastic (NP) flux escaping WWTP barriers [71], as high mass removal efficiencies often mask the release of billions of countable, distinct micro- and nanoscale particles [11,51].

### 5.2. Proposed Dual-Metric Framework for WWTP Monitoring

To overcome the limitations of isolated reporting, we propose adopting a dual-metric framework that integrates spectroscopic identification with thermoanalytical quantification. By combining particle-count data for larger MPs—essential for source tracking and polymer identification [70]—with mass-based metrics for the sub-micrometer fraction—essential for quantifying the total polymer load [73]. Thus, researchers can achieve a more accurate mass balance. This standardized approach is critical for the field’s transition from simple “removal” metrics to an integrative evaluation of toxicity reduction and polymer mineralization (Section 7).

## 6. In-Plant Transformation, Removal Dynamics, and Environmental Risks

### 6.1. Micro-to Nano-Scale Plastics in WWTPs: In-Plant Transformation and Treatment Limitations

Although WWTPs are often reported to achieve high overall removal efficiencies for MPs, accumulating evidence shows that these processes do not ensure the complete elimination of plastic particles, particularly at smaller size scales [6]. Conventional performance assessments have focused on particles in the micrometer-to-millimeter range, whereas finer MPs and NPs (typically < 1 µm) remain undercharacterized due to analytical limitations and the need for high-resolution methods such as Raman microscopy and mass-based thermal techniques [61,70,74]. Recent experimental and full-scale studies indicate that WWTPs function not only as barriers for larger MPs but also as reactors where particle size distributions are modified, with important implications for downstream environmental release and risk redistribution [6,61,71].

Several experimental investigations have demonstrated that the physical and operational conditions commonly encountered in WWTPs, including hydraulic shear during pumping and mixing, aeration, and mechanical handling of sludge, can promote the fragmentation of larger MP particles into smaller size fractions. Laboratory-scale studies under controlled hydrodynamic shear have shown progressive size reduction in polymer particles, generating fragments approaching the nanometer scale [61,71,75]. These findings support the hypothesis that treatment processes designed to remove suspended solids may simultaneously contribute to the formation of smaller plastic particles, which are more difficult to capture using conventional separation mechanisms. As a result, apparent reductions in total MP abundance may be accompanied by shifts toward finer size classes with greater mobility and persistence [61,71,75].

Field observations from full-scale WWTPs further demonstrate that the removal efficiency is strongly size-dependent [6,61,76]. For instance, a recent multi-plant study in Chengdu reported high removal of coarse particles, while the relative abundance of fine fractions (<0.2 mm) remained high in secondary effluent and sludge, indicating much lower effective removal of small particles and potential in-plant fragmentation [75]. Complementary high-resolution analyses of treated effluent have detected nano- and submicrometer plastic particles (roughly 50–2500 nm) in both influent and final effluent, confirming that the smallest fractions can bypass conventional treatment even when overall performance appears high [61,71].

Together, these findings highlight a critical limitation of conventional interpretations of MP removal efficiency. While WWTPs can effectively reduce the mass and number of larger MPs discharged into receiving waters, smaller MPs and NPs may persist in the treated effluent or become concentrated in sewage sludge. This size-selective behavior underlines the need to re-evaluate treatment effectiveness from a system-level perspective that considers particle transformation, analytical blind spots, and post-treatment fate, rather than relying solely on aggregate removal percentages [6,61,71,75], as detailed in Table 8.

The persistence of fine MPs and NPs after wastewater treatment raises important questions regarding their post-effluent fate and environmental implications. Unlike larger MP particles, which are efficiently retained by sedimentation and filtration processes, smaller particles exhibit colloidal behavior that enables their transport through treatment barriers and into receiving waters. Field-based measurements of treated effluent have demonstrated that nano-scale and sub-micrometer plastic particles can remain detectable after conventional and advanced treatment processes, indicating that high overall removal efficiencies do not necessarily translate to the complete elimination of the smallest size fractions [61,71,74,75]. Once discharged, these fine particles may remain suspended in the water column for extended periods, increasing their potential for downstream transport and bioavailability [71,75]. Their small size enhances interactions with natural colloids, organic matter, and microorganisms, which may further influence aggregation behavior, sedimentation dynamics, and trophic transfer [71,75,76].

In parallel with effluent release, numerous studies have identified sewage sludge as the primary sink for MPs within WWTPs, including fine and fragmented particles [6,75,76,77,78]. Size-selective removal processes favor the retention of larger MPs in primary and secondary sludge, whereas smaller particles may be partially retained through flocculation, biofilm attachment, or incorporation into sludge matrices during biological treatment [6,75,76,77,78]. Experimental analyses of sludge samples consistently report substantially higher MP abundances relative to treated effluents, underscoring the role of sludge management as a critical control point in determining the ultimate environmental fate of plastic particles [6,76,77,78,79].

Thus, the land application of treated sludge and biosolids represents a secondary pathway for the environmental redistribution of micro- and nano-scale plastics, reintroducing these contaminants into terrestrial and aquatic cycles [7,52,77,80,81]. This delayed-release mechanism highlights a trade-off inherent to current wastewater treatment strategies: while aquatic emissions may be reduced, plastic particles are often transferred to terrestrial compartments rather than being fully removed from the environment [7,52,77,80,81].

### 6.2. Persistence and Size-Selective Distribution in Effluents

The environmental implications of MPs’ WWTP effluents are significant, as these particles can persist in aquatic ecosystems, bioaccumulate in organisms, transport chemical contaminants, disrupt habitats, and pose potential human health risks [4,7,8,52].

1. Persistence in Aquatic Ecosystems: MPs released through treated effluent tend to accumulate in freshwater and aquatic environments [4,7,8,61]. Their resistance to biological degradation allows them to persist for extended periods, thereby contributing to long-term contamination [23]. In addition, MPs discharged into surface waters can persist in water columns and sediments, with continued detection across diverse aquatic systems [4,23]. Persistent MP debris also interacts with organisms across marine and freshwater ecosystems, influencing ecological processes and contributing to sustained environmental impacts [4,23].
2. Chemical Contaminant Transport: MPs readily sorb pollutants like pesticides, heavy metals, and hydrophobic organic compounds, serving as carriers that enhance the movement and bioavailability of these toxic substances within aquatic systems [12,36].
3. Bioaccumulation and Trophic Transfer: Aquatic organisms may ingest MPs, resulting in their accumulation within tissues and potential transfer along food webs. This process heightens the exposure risks for higher trophic levels, including humans [39,82,83,84].
4. Limited Removal Efficiency: Although advanced WWTP technologies can reduce MP concentrations, complete elimination remains challenging. Residual particles often bypass treatment processes, leading to continuous discharge into receiving water bodies [4,5,50,52,85].
5. Habitat Disruption: The buildup of MPs within sediments can modify the physical characteristics of aquatic habitats, thereby influencing benthic communities and disturbing the stability of the surrounding ecosystem [23,39,47,86].

### 6.3. Retention and Accumulation of MPs in Sewage Sludge

Sewage sludge serves as the primary sink for MPs (80–90% capture), creating significant environmental management challenges. When this sludge is applied to agricultural fields as a fertilizer or disposed of in landfills, it becomes a hidden pathway for MPs to infiltrate soils and groundwater, disrupting ecosystems [87]. These persistent pollutants threaten soil health, harm microorganisms, and can bioaccumulate in terrestrial food webs, posing risks to wildlife and human health. Addressing this issue requires urgent attention to reduce sludge-related MP pollution and promote sustainable waste management practices that protect ecosystems and support environmental resilience [88].

#### 6.3.1. Mechanisms of Sludge Accumulation

MPs are persistent contaminants that enter WWTPs through multiple pathways, including domestic effluents, personal care products, synthetic textiles, and discharge from industrial sources. During treatment, MPs are removed by physical, chemical, and biological processes that separate particulate contaminants from wastewater. Nevertheless, the incomplete elimination of MPs during primary and secondary treatment processes results in a considerable proportion being retained within sewage sludge [40,89]. This is primarily due to their tendency to adsorb onto organic matter and settle during sedimentation [40]. Consequently, WWTPs inadvertently act as both sinks and conduits for MPs, raising concerns about the downstream implications of sludge disposal or reuse, particularly in agricultural applications, where MPs may re-enter terrestrial and aquatic environments [77]. Therefore, learning more about the pathways and behavior of MPs within WWTPs is essential for formulating effective mitigation strategies and guiding policy decisions on sludge management.

#### 6.3.2. Ecological Risks of Sludge Disposal

The reuse of treated sludge in agriculture, which is advantageous for nutrient enrichment, also introduces the potential risk of MP contamination in terrestrial environments. When sludge containing MPs is applied as a fertilizer, these persistent particles become integrated into the soil matrix, where they can remain and accumulate over extended periods. Their presence can modify the physical characteristics of the soil by influencing parameters such as porosity and moisture retention capacity, thereby impairing root development and microbial functioning. Moreover, MPs can adsorb and mobilize hazardous compounds, including heavy metals and hydrophobic organic contaminants, thereby increasing their transport potential and bioavailability within soil systems [90]. These interactions may disrupt the soil microbial balance, interfere with nutrient cycling, and eventually diminish crop productivity.

The prolonged presence of MPs in agronomic soils has raised concerns regarding soil fertility, food safety, and the overall sustainability of land management practices. Over time, MPs from sludge-amended soils can migrate into groundwater or surface water bodies via runoff, thereby extending their environmental footprint and contributing to broader ecological contamination [46,87].

#### 6.3.3. Associated Human Health Risks

The presence of MPs in agricultural soils has emerged as a pressing environmental concern, primarily because crops can take them up and subsequently enter the food supply chain [45,91]. Experimental evidence indicates that MPs can infiltrate plant root systems and migrate to edible tissues, such as fruits and vegetables [91]. The accumulation of MPs in agricultural produce raises serious concerns for human well-being, as ingestion may cause gastrointestinal irritation, weaken immune responses, and facilitate the entry of toxic pollutants such as heavy metals and persistent organic contaminants (POCs) [92].

### 6.4. Environmental Re-Entry Pathways and Global Transport

WWTPs are critical control points in the management of MP pollution, functioning simultaneously as barriers and conduits within the broader environmental system. WWTPs are effective in capturing a considerable fraction of MPs through primary and secondary treatment processes, primarily via sedimentation and aggregation with organic matter [52]. However, these facilities are not explicitly designed to eliminate MPs, resulting in the continued release of residual particles through treated effluent and the transfer of substantial MP loads into sewage sludge. The treated effluent can introduce MPs into aquatic ecosystems [93], whereas the land application of sludge for agricultural purposes advances their accumulation in terrestrial environments. These dual pathways of environmental dissemination underscore the complexity of WWTPs’ role of WWTPs in MP dynamics and highlight the urgent need for improved treatment technologies, standardized monitoring protocols, and regulatory approaches aimed at minimizing MP release at the source and across the treatment continuum [52,93].

#### 6.4.1. Aquatic Dispersion via Treated Effluent Discharge

Aquatic Ecosystem Pollution:

The discharge of treated wastewater from WWTPs into natural aquatic environments, including rivers, lakes, and coastal zones, represents an important pathway for MP contamination to enter freshwater and aquatic systems [93]. Although several treatment stages have been employed, WWTPs are not fully effective in capturing all MP particles, allowing smaller fractions to pass through filtration and sedimentation units. Once discharged, these particles may remain suspended in the water column or gradually accumulate in benthic sediments where they become accessible to a broad range of aquatic species. MPs may be consumed by filter-feeding organisms, zooplankton, benthic invertebrates, and fish, leading to physical and chemical stress, including gastrointestinal blockage, reduced feeding activity, and exposure to adsorbed contaminants [94]. The persistence of MPs, coupled with their ability to accumulate and transfer along trophic chains, amplifies their long-term ecological effects and threatens the stability of aquatic food webs [93,94].

This situation stresses the urgent need for advanced treatment technologies and stricter discharge standards to limit the release of MPs from WWTPs and reduce their environmental impact.

Bioavailability:

MPs detected in treated effluents entering aquatic environments pose a notable risk because they tend to bioaccumulate in living organisms [93]. Once introduced into aquatic systems, MPs are frequently ingested by zooplankton, mollusks, and small fish, which may be mistakenly consumed as a source of food. Because of their minute size and chemical stability, MPs persist within the gastrointestinal system and body tissues of aquatic organisms, potentially causing inflammatory responses, oxidative stress, and behavioral alterations.

As contamination moves upward through trophic interactions, MPs and associated pollutants, such as heavy metals and hydrophobic organic compounds, are transferred along the food web, thereby increasing the exposure risk across species. The adsorptive surface of MPs further enhances their ability to bind hazardous chemicals, intensifying their toxic potential [94]. thereby, this trophic transfer raises serious environmental and health concerns, particularly regarding the consumption of contaminated seafood. Therefore, understanding how MPs are absorbed, distributed, and transferred across biological systems is essential for evaluating their long-term ecological and physiological implications [95].

Aeolian and Runoff:

Primary mechanisms that transport MPs across environments. Wind can carry lightweight MPs, such as microfibers and film particles, over vast distances and sometimes across entire continents [48,96]. This airborne movement of MPs has been observed primarily in areas with large amounts of plastic waste, where wind erosion can detach particles from landfills, beaches, or agricultural fields and transport them to remote areas. Wind transport is significant in regions with limited rainfall and high evaporation rates, where the absence of vegetation eases the movement of these particles [48].

Surface runoff represents a substantial pathway for transferring MPs from terrestrial landscapes to aquatic environments. During periods of intense rainfall or rapid snowmelt, MPs present on urban, industrial, or agricultural surfaces can be mobilized and conveyed through stormwater networks, eventually reaching rivers, lakes, and coastal systems [44]. Urban runoff plays a similar role in the dispersion of MPs across freshwater and aquatic ecosystems. The extent of this runoff is influenced by land-use patterns, prevailing climatic conditions, and the amount of unmanaged plastic waste. Research has shown that highly urbanized regions, particularly those with extensive impermeable surfaces, contribute disproportionately to the overall flux of MPs within drainage systems [44].

Connectivity and Downstream Dispersal:

The continuous movement of water and associated materials, such as MPs, through interconnected aquatic systems, including rivers, streams, estuaries, and oceans. This connectivity is a primary driver of MP dispersal from terrestrial and freshwater environments into aquatic ecosystems [97]. Once released into surface waters through urban runoff, wastewater discharge, or agricultural drainage, MPs can be transported downstream by riverine flow, reaching distant ecosystems and contributing to regional and global plastic pollution [98].

The relocation of MPs through this transport primarily occurs via physical displacement. Lightweight polymers, such as polyethylene (PE) and polypropylene (PP), typically float and remain suspended, enabling their transport over long distances, whereas heavier or biofouled particles may intermittently settle and resuspend based on water velocity and turbulence [99]. For instance, studies in China’s urban estuaries have shown how storm events can intensify downstream MP transport, increasing their concentrations in brackish and aquatic zones [100]. In some cases, hydrological connectivity indirectly mitigates MPs in localized environments by moving them out of high-concentration zones. However, this “removal” is ecological displacement rather than elimination and often results in contamination of more sensitive or remote habitats [99,101]. For example, MPs released in inland cities may accumulate in estuarine mudflats or coral reef systems, where they can affect biodiversity and trophic interactions [98,100,101,102].

Sediment Entrapment:

Once in aquatic environments, MPs frequently undergo settling and entrapment in bottom sediments. This occurs through gravitational settling, especially for denser polymers (e.g., PVC, PET) or MPs attached to organic particles and biofilms. Sediment layers in rivers, lakes, and coastal zones act as temporary sinks, reducing MP concentrations in the water column [99]. In depositional zones, such as deltas, harbors, and estuaries, MPs are buried and can remain stored for extended periods [103].

The mechanism of removal involves adsorption and incorporation into sediment matrices. MPs become entangled with fine sediments, organic matter, and microbial biofilms, thereby increasing their weight and facilitating their settling. However, this process is reversible. Environmental disturbances such as floods, dredging, or biological activity (e.g., bioturbation by benthic fauna) can resuspend MPs and return them to the water column [104]. For instance, in the Paraná River floodplain in Argentina, flood events have been observed to remobilize sediment-bound MPs, leading to episodic contamination surges in downstream aquatic zones [103].

Although sediment entrapment can be viewed as a passive retention mechanism, it also presents an environmental risk. Accumulated MPs may become bioavailable to sediment-dwelling organisms, potentially entering benthic food webs and undergoing trophic transfer [97]. Because sediments are often used as environmental indicators, the presence of MPs in benthic layers reflects both historical pollution trends and ongoing deposition rates.

#### 6.4.2. Terrestrial Contamination via Agricultural Sludge Application

Soil Contamination:

These MP particles tend to remain stable within the soil matrix, altering its physical and chemical characteristics and potentially influencing both soil biodiversity and overall fertility, as demonstrated in studies examining sewage sludge–derived MPs and their persistence following land application [77,89].

**Figure 3:**
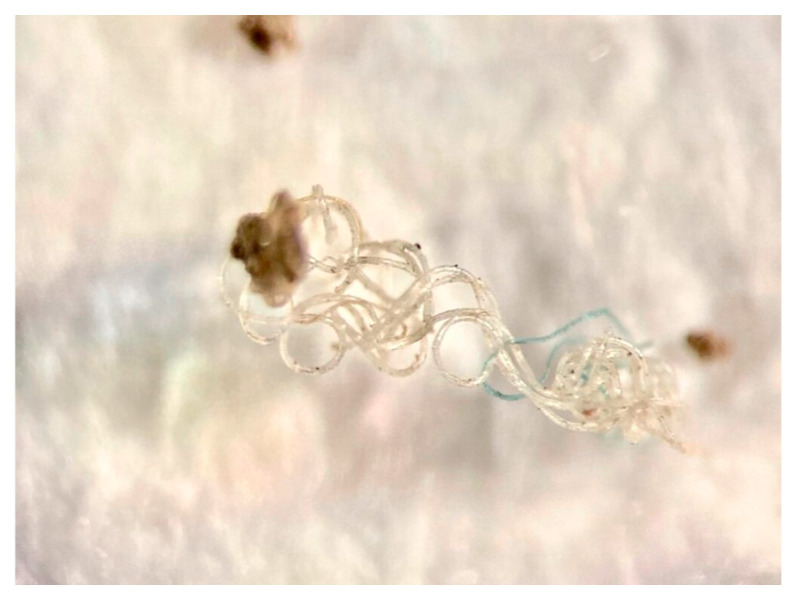
Representative MP fiber morphology commonly observed in environmental samples. Image adapted from the James Hutton Institute [105].

**Figure 4:**
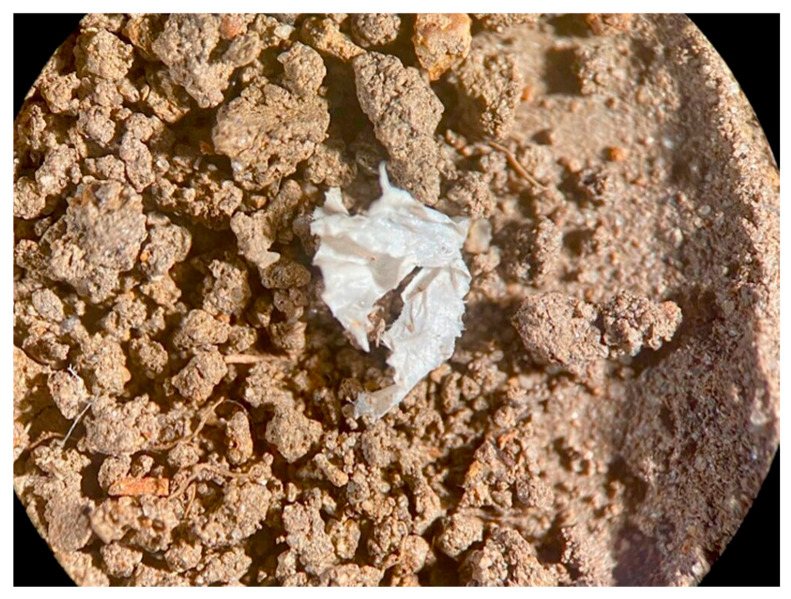
Representative MP fragments embedded in a soil matrix. Image adapted from the James Hutton Institute [105].

Transport to Water Systems:

MPs in the soil may be transported to nearby water bodies via runoff during rainfall, leach into groundwater, or re-enter surface waters through erosion and drainage processes [77,87,89]. This mobility is often influenced by the particle shape and degradation state. Figure 3 and Figure 4 illustrate the typical morphologies of MPs recovered from sewage sludge–amended soils, highlighting the fibrous and fragmented structures that assist both soil retention and subsequent remobilization [105].

#### 6.4.3. Vector Transport of Chemical Contaminants

Pollutant Adsorption: MPs function as highly effective sorbents that bind a range of hazardous contaminants, including pesticides, heavy metals, and persistent organic pollutants (POPs) [35,106]. Owing to their extensive surface area and strong affinity for non-polar compounds, MPs readily attract and retain pollutants from surrounding water, air, and sediment matrices [35,106]. This adsorption capacity allows them to act as carriers that facilitate the mobility of toxic substances across diverse environmental media over considerable distances, thereby amplifying their ecological impact.

For example, MPs in aquatic environments can adsorb heavy metals such as mercury (Hg) and lead (Pb), as well as pesticides and pharmaceutical residues [35,106,107]. The adsorptive capacity of MPs varies with their size, polymer type, and surface properties; PE and polystyrene (PS) are particularly effective at adsorbing hydrophobic chemicals due to their non-polar characteristics [108]. These potential to act as chemical vectors has raised concerns regarding the biomagnification of associated pollutants and the resulting ecological harm, particularly in aquatic food webs where MPs are frequently ingested and transferred across trophic levels [97,108].

Desorption Dynamics: While MPs are efficient at adsorbing harmful chemicals, they also pose a significant risk because adsorbed pollutants can desorb under certain environmental conditions, reintroducing these contaminants back into ecosystems. Desorption dynamics refers to the process by which pollutants, once attached to the surface of MPs, are released back into the surrounding environment when environmental conditions change. This phenomenon is particularly problematic in aquatic systems, where shifts in temperature, salinity, and pH, and biological activity can trigger the desorption of pollutants from MPs [109].

For instance, it has been demonstrated that MPs exposed to varying environmental gradients, such as changes in salinity or the presence of organic matter, exhibit significant desorption of pesticides and hydrophobic organic contaminants [108]. The reintroduction of chemicals into the water column can exacerbate pollution levels, degrade water quality, and increase the bioavailability of toxins to aquatic organisms [97]. Additionally, MPs exposed to bioturbation, the physical disturbance of sediments by organisms such as worms and crustaceans, may experience enhanced pollutant desorption as these organisms move through the sediment and interact with plastic particles, thereby redistributing contaminants.

#### 6.4.4. Global Distribution and Ecological Sinks

Ocean Currents and Long-Distance Transport:

Once MPs are discharged into the environment, they can be transported over vast distances, especially in aquatic systems. Ocean currents play an essential role in the global dispersion of MPs, as these particles can remain suspended in the water column for extended periods and are carried by the dynamic circulation of the ocean. As MPs are relatively light and often buoyant, they can travel across entire ocean basins, accumulating in areas far from their sources, including polar regions and deep-sea ecosystems [110]. The Great Pacific Garbage Patch, a notorious area of concentrated plastic debris, is one of the best-known examples of how ocean currents can accumulate large amounts of MPs over time.

MPs pose a significant environmental risk in remote areas, where they can be ingested by aquatic organisms, potentially leading to bioaccumulation and trophic transfer. For example, zooplankton, the foundation of many aquatic food webs, have been shown to ingest MPs, with the particles potentially transferring up the food chain to larger organisms such as fish, aquatic mammals, and seabirds [97]. The ability of MPs to spread across vast areas and persist in the environment underscores their potential for long-term ecological harm.

MPs in WWTPs:

WWTPs act as major hotspots for MP dissemination worldwide, including those handling effluents and sludge, which are significant conduits for the distribution of MPs into aquatic and terrestrial ecosystems. When treated effluent is discharged into rivers, lakes, or oceans, MPs remain in the water and persist in the environment. In many cases, these particles enter aquatic ecosystems, where they can contribute to bioaccumulation, particularly in aquatic species that ingest MP particles [97].

Moreover, sludge containing MPs is often used as a fertilizer in agricultural practices. This practice introduces MPs into soils, where they may alter soil properties and potentially affect plant growth and soil fauna [111]. MPs may also enter the food chain through crops that absorb contaminated water or via ingestion by terrestrial animals that feed on plant materials or soil organisms [112]. Hence, the transport of MPs through wastewater systems and their deposition in soil ecosystems emphasizes the widespread impact of plastic pollution beyond aquatic environments.

MPs as Vectors for Harmful Chemicals:

MPs can also act as vectors for harmful chemicals, further amplifying their environmental and ecological risks. Due to their high surface area and hydrophobic nature, MPs can adsorb a wide range of toxic pollutants, such as heavy metals, pesticides, and persistent organic pollutants (POPs), during their transport in aquatic environments [36]. These chemicals adhere to the surface of MPs, enabling the particles to serve as vehicles for long-distance chemical transport. Once in remote regions, MPs can release these contaminants via desorption as environmental conditions such as temperature, salinity, or pH change. This phenomenon is a significant concern for ecosystems, as pollutants can be introduced into vulnerable environments, where they can affect organisms across multiple trophic levels [30,36].

Removal Mechanisms for MPs:

Even though the persistence and transport of MPs across environmental media are significant concerns, mechanisms exist that help remove MPs from water bodies and terrestrial environments. In wastewater treatment processes, MPs are predominantly captured during the secondary and tertiary stages using techniques such as coagulation and flocculation. In these methods, coagulants, such as alum, promote the aggregation of MPs with suspended particles, thereby enhancing their separation and eventual removal via sedimentation or filtration [59]. Membrane filtration technologies and sand filters also help capture MPs, particularly in the final stages of treatment, thereby further reducing their release into water bodies [50].

However, the removal of MPs from sludge or soil is challenging. In some cases, MPs may be removed from soil by mechanical processes such as soil washing, which employ mechanical agitation and aqueous or chemical extraction solutions to loosen particle–pollutant associations, thereby facilitating the detachment of MPs and associated contaminants from soil particles.

Despite these interventions, the persistence of MPs in both aquatic and terrestrial ecosystems, coupled with their desorption dynamics, indicates that more effective removal strategies are needed [30].

## 7. Integrated Strategies for Removal and Elimination of MPs

### 7.1. Optimization of Physical and Biological Removal Processes

#### 7.1.1. Advanced Filtration and Membrane Technologies

WWTPs serve as vital interception hubs, capturing MPs before discharge into rivers, lakes, and coasts [50]. Yet conventional processes are limited by MP heterogeneity, low density, and minute size [55].

While standard secondary treatment removes 60–90%, a significant fraction (10–40%) persists [50,51]. necessitating advanced strategies such as MBRs, coagulation, and filtration [51,59].

Membrane Bioreactors (MBRs):

MBRs integrate activated sludge with micro- or ultrafiltration (0.1–0.4 µm pores), trapping MPs with >99% efficiency [50,55]. However, MPs may induce membrane fouling via surface adsorption [57], requiring optimized operational protocols.

Sand Filtration and Ultrafiltration:

As tertiary steps, sand beds physically strain larger fibers (83–99% removal [112]), while UF excludes submicron particles via size exclusion [50,58], significantly enhancing the final effluent quality [113].

#### 7.1.2. Biological Interactions and Plastisphere Management

Biological treatment processes, particularly aerobic and anaerobic digestion, are critical not only for organic matter reduction but also for determining the fate of MPs. Central to this process is the “Plastisphere”—the specific microbial community that colonizes MP surfaces. In the nutrient-rich environment of activated sludge, rapid biofilm formation significantly alters the physicochemical properties of MPs. Recent reviews indicate that biofouling increases particle density and hydrophilicity, driving the sedimentation of low-density polymers (like PE and PP) that would otherwise float, thereby facilitating their partitioning into the sewage sludge line [114].

During anaerobic digestion, microbial interactions intensify. Research has confirmed that digestion conditions induce localized mechanical stress and enzymatic activity, leading to surface weathering. For instance, it has been demonstrated that although anaerobic digestion can reduce the abundance of larger MPs, smaller particles often persist with their surface morphology significantly roughened by microbial activity [115]. Similarly, other studies have found that the presence of MPs can inhibit methane production by affecting the microbial community structure, while the digestion process itself accelerates the detachment of MPs from organic aggregates [116]. More recent work has further elucidated that thermal hydrolysis and anaerobic digestion effectively increase the surface roughness and cracking of MPs, confirming microbial alteration of the polymer matrix [117].

To mitigate this accumulation, bioaugmentation strategies are being explored; specifically, enriching sludge with specific plastic-degrading microbial consortia has been proposed to transform anaerobic digesters into active degradation reactors, thereby reducing the MP load prior to land application [118].

However, the plastisphere poses a critical biological risk. New metagenomic analyses have revealed that MPs in anaerobic digesters act as selective “hotspots” for antibiotic resistance genes (ARGs). Experimental data showed that the plastisphere can enrich ARGs (such as those resistant to tetracycline and beta-lactams) by facilitating vertical and horizontal gene transfer [119]. Furthermore, it has been confirmed that higher dosages of MPs in sludge directly correlate with increased ARG abundance, establishing the plastisphere as a persistent vector of resistance in agricultural soils [120].

#### 7.1.3. Enhanced Chemical Coagulation and Flocculation (C/F)

Chemical C/F is widely employed to destabilize dispersed particulates and neutralize surface charges to promote aggregation. By introducing coagulants such as aluminum sulfate (alum) or ferric chloride, MPs and other colloidal materials form larger, denser flocs that can be efficiently removed via sedimentation or filtration [59,60].

Mechanism and Efficiency:

This process enhances physical separation by increasing the effective particle size of MPs. Research indicates that polyaluminum chloride (PAC) acts effectively on specific fractions, while ferric chloride (FeCl_3_) demonstrates superior removal efficiency across a broader range of particle sizes [61]. For example, Ma et al. [121] reported >80% removal of MPs in drinking water treatment using a coagulation-ultrafiltration sequence. Additionally, investigations into polyester and nylon microfibers confirmed that coagulation significantly improves fiber aggregation, facilitating their capture in subsequent filtration stages [122].

Advantages and Limitations:

The primary advantages of coagulation are its adaptability and ease of integration into existing municipal infrastructure [51,60]. However, optimization is critical; underdosing leads to poor removal, while overdosing generates excessive chemical sludge, increasing disposal costs and potential secondary pollution [52,88]. Moreover, removal efficiency is highly sensitive to water quality parameters (pH and ionic strength) and the specific surface characteristics of the target MPs [60,121].

### 7.2. Transitioning to Elimination: Advanced Oxidation and Electrochemical Technologies

While coagulation and filtration effectively transfer MPs from wastewater to sludge, they do not degrade the polymer matrix. To address this limitation, Advanced Oxidation Processes (AOPs) and Electrochemical Oxidation (EO) are being developed to achieve complete degradation via polymer chain scission.

#### 7.2.1. Advanced Oxidation Processes (AOPs)

AOPs utilize highly reactive hydroxyl radicals (⋅OH) to attack the carbon backbone of MPs, initiating fragmentation and mineralization. Recent experimental work has highlighted the efficacy of Fenton-based systems for treating persistent polymers. For instance, a 2025 study on a magnetite-activated Electro-Fenton system achieved a 90.6% degradation rate for PE MPs within 20 h by cleaving C–C bonds and converting the plastic into low-molecular-weight organics [123]. Similarly, ozonation has proven effective as a pre-treatment for structural alteration; recent data indicate that ozone exposure can cause up to 26.7% surface mass loss in PE particles, increasing their hydrophilicity and making them more susceptible to subsequent biological degradation [124]. However, researchers caution that aggressive oxidation can inadvertently generate NPs through fragmentation if the process is not fully optimized for complete mineralization [125].

#### 7.2.2. Electrochemical Oxidation and Sensing

Electrochemical technologies offer a robust solution to recalcitrant microfibers and particles that can evade traditional treatment. In these systems, MPs are degraded at the anode surface through direct electron transfer or indirect oxidation via the generated reactive species. A breakthrough 2024 study utilizing a TiO_2_-modified Boron-Doped Diamond (BDD) photoanode demonstrated an 89.9% degradation efficiency for high-density polyethylene (HDPE) MPs after 10 h of treatment [126].

Furthermore, the integration of sustainable materials into electrochemical systems has expanded their utility to include real-time monitoring. For example, Kim et al. [127] successfully developed a sensor electrode using naturally manufactured biochar to achieve precise electrochemical detection of polystyrene (PS) MPs. This dual potential for both pollutant degradation and high-sensitivity sensing makes electrochemical systems a cornerstone for future WWTPs, ensuring that MPs are destroyed before they can be released into agricultural soils via sludge application.

### 7.3. Digital Integration: Artificial Intelligence and Machine Learning Applications

The inherent complexity of identifying and quantifying MPs within heterogeneous matrices, including sewage sludge, has necessitated the integration of Artificial Intelligence (AI) and Machine Learning (ML) technologies. These digital tools are currently revolutionizing the field by addressing critical “data gaps” in automated detection and the modeling of environmental transport fluxes.

#### 7.3.1. Automated Detection and Morphological Classification

Manual quantification of MPs is historically labor-intensive and susceptible to observer bias. Recent advancements in Deep Learning, specifically the implementation of Convolutional Neural Networks (CNNs), have advanced the automated classification of MP morphologies, including fibers, fragments, and beads, from microscopic and spectroscopic datasets. Advanced architectures, such as Mask R-CNN, have demonstrated the capacity to process hyperspectral imagery of sludge-derived MPs with high precision, effectively filtering signal noise associated with organic biofilms [128]. Such automation enhances analytical throughput and provides standardized, reproducible data necessary for longitudinal environmental monitoring.

#### 7.3.2. Predictive Modeling of Transport Fluxes

Predicting the migration of MPs from sludge-amended agricultural soils into deeper soil horizons or groundwater remains a significant challenge due to the stochastic nature of rainfall and varying soil porosities. ML algorithms, including Random Forest (RF) and Extreme Gradient Boosting (XGBoost), are increasingly utilized to model these transport fluxes. By training on multi-dimensional environmental datasets, these models can forecast MP accumulation “hotspots” and vertical leaching behavior with a high degree of statistical confidence, serving as essential instruments for environmental risk assessment and the formulation of targeted mitigation strategies [129].

### 7.4. Policy Frameworks and Regulatory Measures

While advanced treatment technologies offer end-of-pipe solutions, the sustainable management of MPs in sewage sludge requires robust policy intervention and source control. Currently, a significant legislative gap exists in global biosolid management. Major regulatory frameworks such as the EU Sewage Sludge Directive (86/278/EEC) [130] and the U.S. EPA Part 503 Rule [131], enforce strict limits on heavy metals and pathogens, but lacks legally binding standards for MP concentrations in sludge applied to agricultural land [51]. This regulatory void effectively permits the transfer of vast quantities of synthetic polymers into terrestrial ecosystems.

#### 7.4.1. Establishing Discharge and Pre-Treatment Standards

To bridge this gap, policy frameworks must shift from general wastewater guidelines to specific sludge quality assurance. Governments should implement discharge limits that classify MPs as priority pollutants, similar to the current regulations for heavy metals. Furthermore, mandating industrial pre-treatment standards is critical. Industries identified as high-emission sources, specifically textile manufacturing and plastic production, must install on-site filtration systems before discharging effluent into municipal sewer networks. This “source control” approach prevents the initial contamination of the sludge line, reducing the burden on municipal WWTPs [114,132].

#### 7.4.2. Extended Producer Responsibilities (EPR) and Standardization

Regulatory bodies can leverage Extended Producer Responsibility (EPR) schemes to drive upstream innovation. By holding manufacturers financially accountable for the end-of-life impact of their products, EPR incentivizes the redesign of textiles to minimize fiber shedding and the replacement of synthetic microbeads with biodegradable alternatives [6,44]. However, the enforcement of such policies relies on the development of standardized analytical protocols. The current lack of harmonized ISO methods for quantifying MPs in sludge hampers regulatory oversight. Therefore, funding and policy support must prioritize the establishment of global monitoring standards to ensure data comparability and compliance [115].

### 7.5. Challenges in Standardization and Ecotoxicological Risks of Degradation Intermediates

#### 7.5.1. The Mass-Number Dichotomy and Nanoplastic Detection

The lack of a standardized reporting metric presents a critical barrier for accurately evaluating treatment efficacy. Recent studies have reported MP removal efficiency using either particle number (items/L) or mass (mg/L), which can yield seemingly contradictory performance data. For instance, a recent full-scale study utilizing pyrolysis–GC–MS across wastewater treatment plants demonstrated that although total MP mass removal exceeded 93%, the removal efficiency of NPs (0.01–1 µm) was significantly lower, resulting in a persistent NP fraction in the final effluent [61]. These finding highlights that even when bulk mass removal appears effective, WWTPs can still discharge substantial nanoplastic loads that remain underestimated if only the total mass is reported.

Furthermore, recent interlaboratory comparisons (2025) have revealed that spectroscopic particle-counting methods frequently fail to detect fractions <10 µm owing to optical resolution limits, whereas thermal degradation techniques successfully quantify these otherwise “invisible” polymer masses [133]. To ensure that elimination is genuine, rather than merely a shift toward smaller, unmonitored fractions, future assessment protocols must adopt a dual-metric approach that combines both particle count and mass-based quantification.

#### 7.5.2. Toxicity of Degradation By-Products

Although advanced degradation technologies offer the potential for polymer mineralization, experimental evidence suggests that incomplete degradation can generate hazardous intermediates. Recent in vitro toxicity trials (2024) have shown that “aged” and partially oxidized microplastic fragments induce significantly higher oxidative stress (e.g., lipid peroxidation) in aquatic organisms compared to pristine polymers [134]. This increased toxicity is attributed to the leaching of monomers and the formation of surface oxygenated functional groups (e.g., carbonyls, hydroxyls) during the oxidation processes [134,135]. Consequently, the objective of WWTPs must shift from simple “plastic removal” to “toxicity reduction.” The proposed solutions to mitigate this risk include incorporating biological polishing steps to degrade hazardous intermediates and mandating whole-effluent toxicity (WET) testing along with standard chemical monitoring [136]. However, the widespread implementation and commercial viability of these advanced solutions still require further development and investment.

## 8. Conclusions

MPs have emerged as ubiquitous environmental contaminants, with WWTPs acting as both primary interception points and unintended gateways for their release. As this review has demonstrated, current treatment trains are effective at partitioning larger MPs into sewage sludge, but struggle to contain the fine fraction and NPs formed through in-plant fragmentation. Consequently, while effluent quality may appear high based on mass removal, the pollutant load is merely transferred to biosolids, creating a significant secondary pollution pathway when sludge is applied to agricultural soils.

To resolve this trade-off, the wastewater sector must transition from simple physical separation to complete mineralization and toxicity reduction. Emerging technologies such as AOPs and Electrochemical Oxidation have proven capable of mineralizing recalcitrant polymers while mitigating the risk of hazardous intermediates, offering a definitive solution to preventing soil contamination. Furthermore, the “methodological gap” identified in this study is being addressed by Artificial Intelligence (AI). The integration of deep learning for automated morphological classification and machine learning for predicting transport fluxes offers a standardized framework necessary for future monitoring and risk assessment.

However, technological optimization alone is insufficient. A holistic management strategy requires filling the current legislative void in biosolid management. Urgent policy action is needed to establish legally binding sludge quality standards for MPs, mirroring those for heavy metals. When combined with upstream source control, such as industrial pre-treatment and Extended Producer Responsibility (EPR). These multi-dimensional strategies provide a sustainable roadmap for mitigating the environmental footprint of wastewater infrastructure. Finally, it must be acknowledged that the findings of this review are constrained by the significant heterogeneity and reporting inconsistencies in the current MP literature. Addressing these data gaps through our proposed dual-metric framework is essential to ultimately safeguard both aquatic and terrestrial ecosystems.

## Figures and Tables

**Table 1:** Morphological Classification and Physical Characterization of MPs.

Type of MP	Typical Size	Origins	Characterization	References
Fragmentations	1 µm–5 mm	Packaging, containers, and household waste	Irregular debris resulting from in situ fragmentation or surface attrition of bulk polymer materials.	[4,11,12]
Pellets	1 mm–5 mm	Plastic manufacturing industry	Pre-production industrial resins (nurdles) are typically introduced by accidental spillage during transport or site runoff.	[12]
Fibers	1 µm–5 mm	Synthetic textiles (polyester, nylon)	Anisotropic strands are released during the mechanical agitation of garments in laundering and drying cycles.	[12,13]
Microbeads	50 µm–500 µm	Personal care products (PCPs)	Engineered spherical particles are utilized in exfoliants and cosmetics; they frequently bypass primary treatment due to their low density.	[11,13]
Films	<1 mm–5 mm	Plastic bags, agricultural mulch, and degraded wrapping materials.	Two-dimensional thin-layered flexible sheets with high surface-area-to-volume ratios.	[4,12,13]

**Table 2:** Source Profiles, Physicochemical Characteristics, and Entry Pathways of Primary MPs.

Primary Source	Technical Characterization	Typical Size Range ^1^	References
Personal Care (PCPs)	Engineered Polyethylene (PE) and Polypropylene (PP) spheres are utilized as exfoliating agents in cosmetic formulations.	4.1 µm–1.2 mm	[14,15,16]
Industrial Abrasives	Spherical or angular resin pellets are designed for “air-blasting” operations, paint stripping, and surface finishing.	100 µm–5 mm	[1,12]
Synthetic Textiles	Direct shedding of microfibers from polymer-based garments during industrial laundering or textile processing.	1 µm–5 mm	[4,13]
Medical Applications	Specialized polymer-based delivery systems have been designed for targeted drug release and biomedical diagnostic procedures.	100 nm–1 mm	[17]
Agricultural Inputs	Engineered polymer coatings for controlled-release fertilizers and encapsulated pesticides in precision farming.	10 µm–1 mm	[18,19,20]
Paints and Coatings	Fragments are derived from mechanical abrasion, weathering, or chipping of industrial and marine surface coatings.	1 µm–100 µm	[21]

^1^ Reported size ranges represent a synthesis of representative experimental values and detection limits characterized across the cited literature.

**Table 3:** Origins, Morphological Characteristics, and Size Distribution of Secondary MPs.

Origins	Characterization	Typical Sizes	References
Plastic Degradation	In situ fragmentation of macrodebris driven by photo-oxidative (UV) exposure, mechanical attrition, and chemical weathering of bulk polymers.	1 µm–<5 mm; progressive degradation can yield sub-micrometer fragments.	[22,23]
Textile Fibers	Anisotropic microfibers are released from synthetic polymers (e.g., polyester, nylon) during mechanical agitation in laundering and drying cycles.	1 µm–5 mm; length-to-diameter ratio significantly influences buoyancy.	[24]
Rubber Particles (Tire Wear Particles, TWPs)	Elastomeric particles are generated via tire–road interfacial abrasion; the composition typically includes natural and synthetic rubber (NR, SBR) laden with road dust.	20 µm–500 µm, predominantly detected within the fine fraction of stormwater runoff.	[25]
Paint Fragments	Highly irregular, micron-to-millimeter-scale debris resulting from the structural failure, chipping, and weathering of industrial and marine coatings.	1 µm–100 µm.	[26]
Aquatic Equipment	Direct environmental release of polymer fragments through mechanical degradation of fishing gear, ropes, and marine infrastructure.	50 µm to several mm.	[27]

**Table 4:** Physicochemical properties of MPs govern their fate, transport, and removal in WWTPs.

Property Category	Feature/Parameter	Technical Characterization	Environmental Significance & Impact on Fate	References
Physical Properties				
	Size ^1^	Spans a continuum from macro-debris (>5 mm) to sub-micrometer NPs; <1 µm)	Governs bioavailability, translocation across biological membranes, and capture kinetics within WWTP stages.	[11,23,27]
	Morphology	Heterogeneous shapes (fibers, fragments, films, pellets, foams) resulting from manufacturing origins or environmental aging.	Strongly influences drag coefficients, buoyancy, and mechanical probability of entrapment within bio-flocs.	[11,23,29]
	Density	Polymer-specific property (e.g., 0.89–0.91 g/cm^3^ for PP vs. 1.30–1.58 g/cm^3^ for PVC).	Determines the vertical distribution in the water column; low-density polymers favor effluent discharge, whereas high-density fractions settle into sludge.	[11,12,23,30]
	Color	Ranges from transparent and white to vibrant primary colors (blue, green, red).	Dictate selective predatory ingestion risk, as visually prominent particles are frequently mistaken for prey by aquatic organisms.	[31,32,33,34]
	Specific Surface Area	Inversely proportional to the particle radius increases significantly as MPs fragment into NPs.	Regulates the sorption capacity for hydrophobic pollutants and potential for biofilm colonization.	[30,35]
Chemical Properties				
	Polymer Composition	Dominance of synthetic resins, including PE, PP, PS, PVC, and PET.	Determines the fundamental density and molecular stability of the particle within wastewater systems.	[7,13,23]
	Chemical Additives	Integration of plasticizers, flame retardants, stabilizers, and dyes.	Functional additives may leach into the environment and potentially exert toxic effects on aquatic organisms.	[36,37,38]
	Sorption Capacity ^2^	High hydrophobicity and large surface area facilitate the capture of nonpolar pollutants.	MPs serve as vectors for hazardous contaminants, including PCBs, PAHs, and heavy metals.	[30,35,36]
	Persistence	Exceptional resistance to biological and enzymatic mineralization.	Breakdown occurs primarily via abiotic processes (UV radiation and oxidation) rather than biodegradation.	[23,39]
	Interaction with Contaminations	Dynamics of pollutant adsorption and subsequent release (desorption).	Adsorbed pesticides and pollutants can desorb during ingestion, thereby increasing their bioavailability.	[30]

^1^ Reported size ranges represent a synthesis of representative experimental values and detection limits characterized across the cited literature. ^2^ Sorption capacity and leaching kinetics are highly dependent on environmental factors such as pH, salinity, and the aging degree of the polymer surface.

**Table 5:** Influent Loading and Primary Transport Pathways of MPs to WWTPs.

Pathways ^1^	Characterization	References
Domestic Wastewater	Originates from synthetic textile laundering, personal care product usage, and household cleaning residues.	[13,14,43]
Industrial Effluents	Discharges from manufacturing processes, including resin pellet spills and production of synthetic rubber.	[42,44]
Urban Runoff ^2^	Stormwater conveyance of MPs from road surfaces, tire-wear particles, and mismanaged plastic waste.	[40,41]
Agricultural Runoff	Transport of MPs from fertilizers, biosolids, and mulch films via surface drainage.	[45,46,47]
Atmospheric Deposition	Airborne MPs that settle via dry deposition or precipitation subsequently enter sewer networks.	[48,49]

^1^ The relative contribution of each pathway varies significantly based on regional industrialization, population density, and the presence of combined vs. separate sewer systems (CSS/SSS). ^2^ Urban runoff contributions, particularly tire-wear particles (TWPs), exhibit high temporal variability influenced by “first flush” storm events and traffic intensity.

**Table 6:** Comparative Removal Efficiencies of MPs Across Conventional Treatment Stages and Advanced Technologies.

Treatment Process	Efficiency (%) ^1^	Size/Morphology Target	Mechanism	References
Preliminary + Primary Treatment	32–93% (avg ~72%)	>150 µm; 27–149 µm; majority of fibers	Hydrodynamic screening. Density-driven settling	[4,6]
Secondary (AS, Biofilms) ^2^	~16% (Avg. additional)	106 µm–300 µm; Biofilm-associated MPs	Bio-flocculation Entrapment in activated sludge Biofilm adhesion	[4,6,13]
Combined (Primary + Secondary)	88% (Average)	Broad MP range (all sizes)	Sequential settling Phase partitioning into sludge	[4,6]
MBR (Tertiary)	99.4–99.9%	Fine fractions (<10 µm)	Size-exclusion membrane filtration Cake layer formation	[11,50,51]
Rapid Sand Filtration (RSF) (Tertiary)	83–99%	50 µm–500 µm; Fibers/Fragments	Mechanical straining Depth filtration	[4,11,50,55]
Coagulation-Flocculation	57–100%	1 µm–500 µm; Microfibers	Charge neutralization Sweep-floc formation	[11,51,59,60]
Dissolved Air Flotation (DAF)	~95%	Buoyant MPs (PE, PP)	Bubble–particle attachment, flotation	[11,50]

^1^ Removal efficiencies are primarily reported based on particle count; however, mass-based removal rates may differ significantly due to the presence of large, high-density fragments. ^2^ The “Average additional” efficiency for secondary treatment refers to the fraction of particles removed from the primary effluent, specifically, rather than the total initial influent load.

**Table 7:** Methodological Framework for the Extraction, Identification, and Quantification of MPs in Wastewater Matrices.

Analytical Method	Mechanism & Application	Key Advantages	Limitations	References
Fractioned Filtration	Sequential Stainless steel sieving for initial size-based segregation and volume reduction.	Simple; immediate size segregation.	High risk of clogging; no polymer ID.	[66]
Wet Peroxide (WPO)	Chemical digestion (H_2_O_2_ + Fe catalyst) for isolating MPs from Biogenic organic matter.	Highly effective for removing biogenic interference in sludge/influent.	Potential for structural degradation of resins.	[67]
FTIR Spectroscopy	IR absorption band detection for definitive polymer resin verification and fingerprinting.	Non-destructive; provides definitive polymer fingerprinting (PE, PP, PET).	LOD: ~20 µm, sensitive to water/organics.	[68,69]
Raman Spectroscopy	Inelastic laser scattering for sub-micrometer characterization.	Exceptional spatial resolution; capable of identifying particles < 1 µm.	Sensitive to fluorescence interference	[69,70,71,72]
SEM/EDX	High-magnification electron imaging for surface morphology and elemental additive profiling.	Provides ultra-detailed surface topography and identification of inorganic or metallic additives and surface contaminants.	Requires skilled operation, no polymer ID	[11,71,72]
Py-GC-MS	Thermal degradation for mass-based quantification.	Simultaneous mass quantification of polymers and detection (and in some methods semi-quantification) of associated additives	Relies on irreversible thermal degradation; requires complex external calibration with polymer standards	[51,71,73]

Note: The lower limits of detection (LOD) and quantification (LOQ) are significantly influenced by particle weathering, automated identification accuracy, and fragmentation of MPs into sub-micrometer fractions. Cost and duration estimates vary according to the region and laboratory capacity.

**Table 8:** Size-Dependent Removal Limitations and In-Plant Transformation Mechanisms.

Treatment Scale	Observed Behavior	Implications for MP Transformation & Redistribution	References
Full-scale WWTP	MPs were detected in the treated effluent despite high mass removal; smaller particles showed lower capture rates.	Mass-based efficiency masks the continuous discharge of millions of mobile small-scale particles into aquatic systems.	[13]
Pilot- and full-scale tertiary treatment	Tertiary stages enhance removal, but particles < 100 µm persist consistently in the final effluent.	Advanced technologies face size-exclusion limits, leading to the chronic release of fine MPs into receiving waters.	[50]
Megacity WWTPs	Removal was >85% for MPs > 1 mm, but dropped below 50% for particles < 0.2 mm across all stages.	Treatment shifts the particle size distribution toward smaller, more persistent fractions rather than achieving total elimination.	[75]
Sewage treatment	NPs (10–1000 nm) were detected in treated wastewater using high-resolution analytics.	NPs bypass conventional activated sludge barriers, representing a major but under-monitored exposure pathway.	[71]
Experimental and process-level analysis	Hydraulic shear and mechanical stress promote the fragmentation of MPs during processing.	WWTPs can act as secondary sources of smaller MPs and NPs via internal mechanical degradation.	[74]
Chemical Coagulation (Tertiary)	Tertiary coagulation/flocculation of secondary effluents promotes the rapid transfer of MPs from water to sludge aggregates.	Apparent effluent removal mainly reflects a phase shift from the aqueous phase to sludge, concentrating pollutants for terrestrial redistribution.	[59]

## Data Availability

No new data were created or analyzed in this study. Data sharing is not applicable to this article.
